# Development of an Atmospheric Pressure Chemical Ionization Interface for GC-MS

**DOI:** 10.3390/molecules25143253

**Published:** 2020-07-16

**Authors:** Christian Lipok, Florian Uteschil, Oliver J. Schmitz

**Affiliations:** University of Duisburg-Essen, Applied Analytical Chemistry, Universitaetsstr. 5, 45141 Essen, Germany; christian.lipok@uni-due.de (C.L.); florian.uteschil@uni-due.de (F.U.)

**Keywords:** GC-APCI, plant protection products, coffee analysis, ion source development, GC-APCI-MS

## Abstract

A closed atmospheric pressure chemical ionization (APCI) ion source as interface between a gas chromatograph (GC) and a triple quadrupole mass spectrometer (QqQ-MS) was developed. The influence of different ion source conditions, such as humidity, make-up gas flow, and the position of the GC column, were investigated and determined as main factors to increase sensitivity and repeatability of the system. For a performance test under real conditions, the new APCI ion source was used for the determination of plant protection products in commercially available coffee beans from Vietnam. The ionization behavior was investigated and the majority of the analytes were detected as [MH]^+^, [M]^+∙^, or as characteristic fragment ions, which have been assigned to ion source fragmentation. The developed GC-MS methods are based on tandem MS (MS/MS) and revealed for the plant protection products limits of detection (LOD) between 1 and 250 pg on column and relative standard derivations for all compounds < 16%. The used ultrasonic solid–liquid extraction yielded recovery rates of approximately 60 to 100%. Residues of herbicide methyl esters, organophosphorus compounds, and organonitrogen compounds have been detected in the analyzed coffee beans.

## 1. Introduction

Atmospheric pressure chemical ionization (APCI) was introduced 1973 by Horning et al. [1] as an alternative to the electron impact (EI) ion source. Until now, EI is still the most common ion source for interfacing a gas chromatograph (GC) to a mass spectrometer (MS), because of the high reproducibility of the systems and the ionization process [2]. The applied energy of 70 eV leads to a high grade of fragmentation, and EI is known as a hard ionization technique. This offers the possibility to identify substances by their characteristic fragmentation pattern by comparison with EI databases. Unfortunately, the fragmentation can cause to low detection limits due to similar fragment spectra or coelution to incorrect identifications. The low abundance or even absence of [M]^•+^ makes the identification sometimes difficult or impossible. This is a problem in nontarget analysis of complex samples using GC and, e.g., high-resolution mass spectrometry (HRMS) where the exact mass of the analyte is mandatory [3,4,5,6].

**Figure 1:**
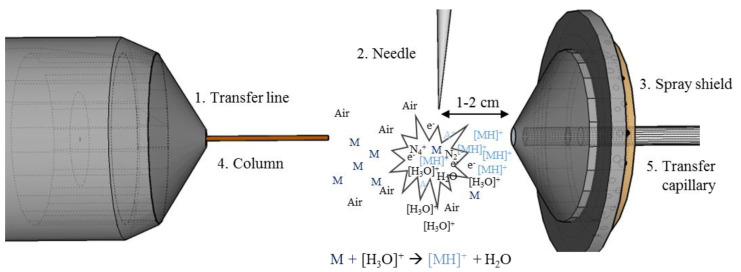
Overview of a conventional gas chromatography-atmospheric pressure chemical ionization-mass spectrometry (GC-APCI-MS) set up. The system contains the transfer line (1), the APCI corona needle (2), spray shield (3), the GC column (4), and a transfer capillary (5). The needle is mounted 1–2 cm from the interface system. Between the needle and the inlet system is the plasma zone, where the reaction cascade take place.

There is the possibility to generate the molecule peak by means of reactant gas in the GC-CI (i.e., in a vacuum). However, the slightly increased pressure in such ion sources, among other things, does not greatly increase the sensitivity. In addition, there are only very few GC-MS instruments on the market that allow high mass resolution. Because of the soft ionization process with almost no fragmentation and the possibility to couple this to very sensitive or high-resolution MS systems, APCI is an alternative for EI. The soft ionization process suits tandem MS (MS/MS) applications, because of the high abundance of the molecular ion. Because of the increased sensitivity and selectivity of MS/MS, it is used more frequently in target screenings and will favor the usage of soft ion sources in the future. [7] Moreover, linked to HRMS, it is possible to obtain high accurate molecular ion information [8]. Despite all these advantages, GC-APCI was never really commercialized [9]. That changed at the beginning of the century with the publications of McEwen et al. [10] and Schiewek et al. [11]. They could interface a GC to an atmospheric pressure mass spectrometer, which were usually coupled to liquid chromatography and improved the versatility of MS systems. Since then, commercial APCI ion sources from different vendors are available. A conventional construction of an open APCI ion source chamber with a corona needle in front of the inlet is shown schematically in Figure 1. Usually, the corona needle is mounted 1–2 cm away from a heated inlet system with a slight offset to the inlet system. This can be either a transfer capillary or a skimmer. The ion source housing is normally open to ambient air or not sealed, and this causes high background signals and irreproducible conditions inside the ion source [12,13]. Therefore, the majority of these APCI sources show relative standard derivations of approximately 10 to 60% [14,15,16,17]. Hence, performance criteria for analytical methods are not always met, and the aim of this work was to develop a more reproducible APCI ion source for GC-MS instrumentation.

The well-described GC-APPI ion source presented by Kersten et al. [18] was used as a basis for the development of the GC-APCI source. The advantages of this closed ion source are high degree of mixing between analytes and reactant ions, closed to ambient air, small ion source volume, exact adjustment of the gas flows.

APCI as a soft ionization technique is commonly used for substances with a high amount of fragmentation and practically no molecular ions under EI conditions. This is in general the case for highly chlorinated compounds and plant protection products [19,20,21,22]. Typical applications of GC-APCI are the determination of these analytes in biological samples such as fruits, vegetables, and meat [23,24,25], or environmental samples such as surface and waste water [26,27,28]. Furthermore, the application of GC-APCI in different fields, such as the determination of terpenes and phenolic compounds in oils for quality control [29], metabolic profiling [30,31], and developments of pharmaceuticals [32,33], has been demonstrated.

The determination of plant protection products in food products is still relevant, as seen by the discussion over the usage of glyphosate or the prohibition of chlorpyrifos in Europe in January 2020 [34]. Monitoring of these substances is mandatory, because of their persistence in the environment and harmful effects on animals and plants. Furthermore, accumulation in the food chain is possible and may cause mutagenic, carcinogenic, and teratogenic effects if consumed [35].

According to the Food and Agriculture Organization of the United Nations, coffee is one of the world’s most exported products with an annual production of approx. 13 million t in 2018 and Vietnam is, since 2012, the second biggest exporter [36]. Therefore, the performance of the developed ion source was investigated on commonly used plant protection products in Vietnamese coffee. In total, 142 compounds with different functional groups (organochlorine OCP, organonitrogen ONP, organophosphorus OPP, methyl esters HME, and synthetic pyrethroide SPP) were investigated. At first, the influence of different ion source parameters, such as humidity and temperature, are described, and secondly, the performance of the developed GC-APCI-coupling is evaluated in terms of LOD, linearity, and recovery rates. The developed method was applied on the determination of residues from plant protection products in a commercially available coffee from Vietnam.

## 2. Results and Discussion

### 2.1. Development of the GC-APCI Ion Source

**Figure 2:**
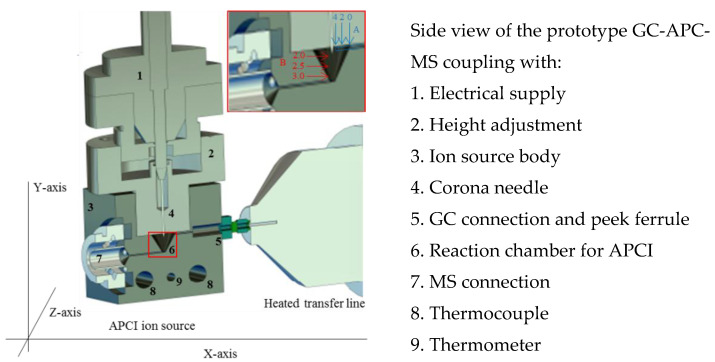
Drawing of the new GC-APCI-MS ion source design and coupling. For the optimization of the column position, the distance between the column and ion source body was set to 0, 2, and 4 mm. This is seen by arrows A. At 0 mm, the column outlet and the ion source wall are at the same hight. Arrow B shows the variation of the corona needle in the *y*-axis, which is given in a distance between the closing plane of the needle holder of the ion source chamber and the tip of the needle.

In conventional GC-APCI setups, the corona needle is positioned in front of the MS entrance and a heated transfer line is guided from the GC side wall to the mass spectrometer entrance. This source chamber design is equipped with an exit port for the excessive gas flows from the mass spectrometer (e.g., drying or curtain gas). This leads to a high background, unstable ion source conditions, and poor repeatability [13]. Therefore, the presented closed ion source design from Kersten et al. was adapted and optimized for GC-APCI [18]. The schematic drawing of the developed ion source is shown in Figure 2. It includes the experimental set up for the variation of the needle height (B, variation in *Y*-axis) and the possible variation of the column position (A, variation in *x*-axis). The developed protype ion source housing is made of stainless steel with a size of 4 × 4 × 4 cm (3). The metal cube holds the APCI needle (4) and high voltage connector (1), which are taken from the commercially available GC-APCI ion source (Agilent Technologies, Santa Clara, CA, USA) and are made from stainless steel and PEEK. The height of the APCI needle can adjusted over the self-constructed needle holder (2) made from PEEK. The GC column was introduced over an opening (5) and closed via a PEEK ferrule. The metal block is temperature regulated over an electrical isolated thermocouple (8–9) and can adjusted between 70 and 300 °C. The reaction chamber for the APCI process (6) had a total volume of 0.12 cm^3^ and is approximately 1000 times smaller than conventional GC-APCI ion sources from commercial manufactures. The ion source body (3) can be directly connected to the transfer capillary (7) of the 6495 triple quadrupole LC/MS and GC (5), both from Agilent Technologies (Santa Clara, USA).

**Figure 3:**
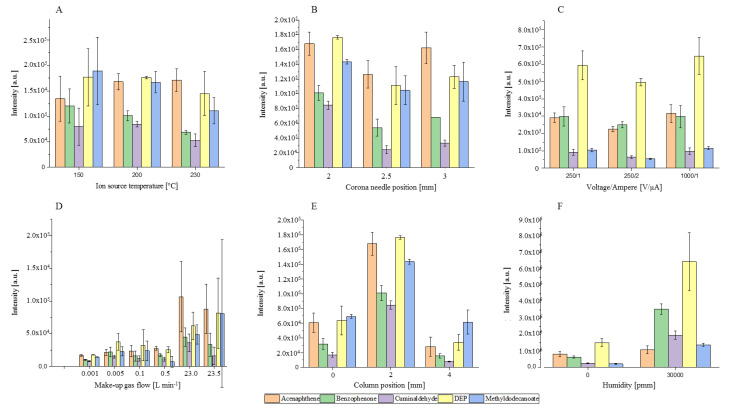
The influence of ion source parameters on the averaged (*n* = 3) peak intensity (a.u.) of the [MH]^+^ ions from acenaphthene (orange), benzophenone (green), cuminaldehyde (purple), diethylphthalate (DEP) (yellow), and methyldodecanoate (blue) as a function of ion source temperature (**A**), corona needle position (**B**), electrical field (**C**), make-up gas flow with high and low pressure region (**D**), column position (**E**), and humidity (**F**). Each parameter is changed separately from the starting method: needle height 2 mm, column position 2 mm, ion source temperature 200, electrical field 1000 V and 1 µA, humidity 0.1 ppm.

To investigate the ionization behavior in this source chamber, important parameters such as temperature (°C), make-up gas flow rate (L min^−1^), column position (mm), needle position (mm), the humidity in the make-up gas flow (water concentration in the gas in ppm (*v*/*v*)) and corona discharge parameters, such as corona needle voltage (V) and corresponding ionization current (µA), were varied. For this, different compound classes that are frequently analyzed by GC were used [37,38]. The above-mentioned ion source parameters and their influence on ion yield and transmission are summarized in Figure 3 A–F. The figure shows variation of the peak height (Intensity (a.u.)) in relation to one of the adjusted parameters.

As shown in Figure 3A–C the ion source temperature, the corona needle height and the electrical field have a minor impact on ionization efficiency and ion transmission. The signal intensity of the [MH]^+^ ions decrease for four of the five substances with increasing temperature. The higher temperature shifts the chemical equilibrium to the deprotonated analyte species and could explain the lower protonation yields [39]. For acenaphthene, a slight increase of the signal intensity and protonation yield is observed. It seems that the reaction rate constant is increased. This is possible because protonation reactions can correspond to thermodynamic or kinetic controlled reactions [40,41,42]. Overall, the influence of the temperature on the ion yield is difficult to predict. On one hand, the reaction rate constants increase with temperature and should lead to a higher reaction yield. On the other hand, the equilibrium of the reaction is shifted to the deprotonated analyte. Additionally, the pressure of the ion source increases with the temperature and hence, so do the collision and reaction rates. Overall, we recommend a heated ion source. The formation of cold spots and adsorption of molecules on the ion source wall is minimized. Furthermore, Sunner et al. [41] demonstrated that the sensitivity of protonated molecules with low water cluster stability improved by several orders of magnitude with increasing temperature. The influence of the needle depth was investigated between 2 and 3 mm and is shown in Figure 3B. At shorter distances, no plasma was generated and hence no ions could be detected. At a distance higher than 3.5 mm, the increased filed strength leads to arcing. Moving the needle tip inside the ion source has no impact on the peak height and hence sensitivity of the system. In addition, a slight decrease in accuracy can be observed when the needle reaches further into the ion source chamber. It seems that the needle has some impact of the flow profile of the make-up gas and formation of the vortex. Figure 3C shows that a changing of the electrical field over current and voltage has no influence. Roesch et al. [14] and Bartosińska et al. [43] reported similar results for the commercial LC- and GC-APCI interfaces from the same instrument vendor. With APCI ion sources from different manufacturers, the same results were obtained [44]. We observed that the voltage of the corona needle and transfer capillary are set in opposite ways from the operating software. An increase in the voltage of the corona needle leads to a decrease in the capillary voltage; therefore, the electrical field gradient inside the ion source remains constant.

As shown in Figure 3D–F, the parameters of make-up gas flow rate, humidity, and column position have a significant influence on the peak intensity and repeatability of the system. Figure 3D shows that the make-up gas is needed to ignite the plasma and hence start the ion–molecule reactions in the ionization region. The sensitivity increases with increasing make-up gas flow and the resulting peak heights were 6 to 11 times higher at 23 L min^−1^ compared to 1 mL min^−1^. The higher pressure in the ion source leads to higher collision rates and hence the reaction rate constants and yields [45]. Protonation reactions are highly efficient and occur on almost every collision if the reaction is exothermic [46]. Unfortunately, the higher flow rates lead to an increase in RSD up to 100%. In this set-up, the make-up gas was controlled over a mass flow controller with a precision of 0.1%. Because of the ionization chamber volume of 0.12 cm^3^, small fluctuation of the regulation can cause high standard derivation. With an accuracy of 0.1%, the mass flow controller has an error of ±23 mL min^−1^, which is 200 times higher than the ion source volume. The column position (Figure 2, Arrow A) had a significant impact on the repeatability and sensitivity of the system. A change of the capillary position from the 2 mm position—in the middle between the needle and the ion source wall—leads to a decrease in sensitivity and increase in RSD. Wall effects become predominant when the column exit port and the ions source wall have the same position (0 mm). At the centre of the ion source (4 mm), the eluent can hit the corona needle. Furthermore, the highest velocities and turbulence are in the centre of the ion source [18].

To increase the humidity in the ion source (Figure 3F), the make-up gas was led through a closed and heated water vessel at a constant temperature of 50 °C. That provided a constant humidity level of higher than 10,000 ppm, which was controlled by a trace moisture analyzer PPM1 Form EdgeTech, in the make-up gas. The peak intensity for the [MH]^+^ ions increased with humidity by a factor of 2 to 9. More reactant ions were formed and available for the ionization process. The probability of collisions between water clusters and analytes was increased and thus the reaction rate [16,47]. Mainly, the intensity of the oxygen-containing compounds had been improved. The peak intensity from acenaphthene was only slightly affected. The lower probability of water–analyte cluster formation may be because of the higher lipophilicity of acenaphthene in comparison to the other compounds. Furthermore, for all compounds, a reduced repeatability was obtained.

**Figure 4:**
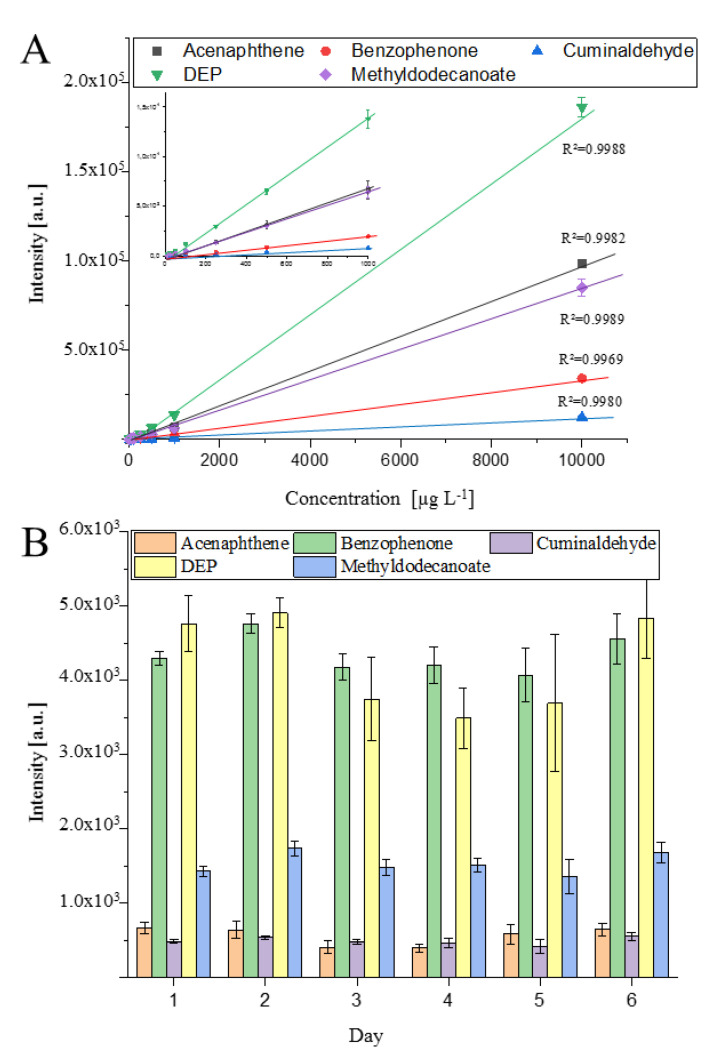
Performance of the prototype ion source. (**A**) shows the linearity and standard derivation of the standards between 10–10,000 µg L^−1^. Each concentration was analyzed three times (*n* = 3). (**B**) shows the repeatability of the measured values over six days with five injections (*n* = 5) at 1 mg L^−1^ each day.

A combination of settings for the highest repeatability—column position 2 mm, ion source 200 °C, dry gas 200 °C, make-up gas 1 mL min^−1^, needle 1000 V and 1 µA, transfer capillary 250 V, humidity 0.1 ppm—was used for evaluation of linear range and inter/intraday precision, which are shown in Figure 4. The behavior of the detector response (Intensity (a.u.) as a function of the concentration (µg L^−1^) is given in Figure 4A. Furthermore, Figure 4B shows the inter/intraday precision over six days with five injections each time.

Over six days, only marginal shifts in the peak height were obtained. The averaged peak intensity of each day showed a normal distribution around the averaged peak intensity over the six days and an RSD for the interday repeatability <15% was obtained for all compounds, except DEP. On average, an intraday repeatability with RSD values <10% was obtained. Only at day 5 of the series of measurements did higher standard derivations of approximately 20% occur. Normally, RSD values <7% were obtained as seen by the calibration curve in Figure 4A. The RSD values were for each concentration and analyte were < 7%. A linear relationship between the peak height and the concentration was found between 10 and 10,000 µg L^−1^ with a correlation coefficient of R^2^ >0.997. No detector saturation was observed, and a greater linear range can be expected for these compounds.

LODs were determined using the 3σ method based on Kaiser and Specker [48] and are shown in Table 1.

LODs between 0.5 and 2.5 pg on column, which were obtained for the used standards with the developed prototype APCI source, are in the same order of magnitude as commercial APCI ion sources even under high precision and lower sensitivity conditions [9,14,49,50,51]. However, a comparison is difficult due to the different proton affinities of the analytes and hence different sensitivities [41,42].

### 2.2. Target Analysis of Plant Protection Product Residues in Coffee Beans

To demonstrate the capabilities of the developed GC-APCI-QqQ-MS coupling, 142 plant protection products from four different classes were investigated. These compounds were chosen because of their important role in food safety and usually low concentration in real samples. In total, 123 compounds were detected as [MH]^+^, [M]^+^, or characteristic fragment ions such as [M − Cl]^+^, [MH − Cl]^+^, and [MH − H_2_O]^+^ using the developed GC-APCI ion source. An increase in sensitivity was observed for more than 90% of the used plant protection products with additional humidity (S2A-G). For some polychlorinated compounds, decreased sensitivity was observed. These compounds are ionized by charge transfer, and increased humidity will decrease this process in the ion source [9,14,16,52]. Unfortunately, RSD values also increased with the humidity; therefore, the analyses were performed without the addition of extra water to the make-up gas. The fragmentation patterns of all substances were investigated by 10 collision energies between 5 and 70 V, and the product ions with the highest intensities were further used for the dMRM methods (Supplementary Table S2). These methods were used for targeted screening of the plant protection groups in Vietnamese coffee beans. As shown in Table S2, the liquid–solid extraction leads to an average extraction efficiency of 57% for organophosphorus compounds (OPP), 105% for herbicide methyl esters (HME), 75% for synthetic pyrethroid compounds (SPP), and 108% for organonitrogen compounds (ONP). For OPP and ONP compounds, lower extraction efficiencies were observed, because these substance classes have further functional groups and changed polarity. For example, 30 of 40 OPPs showed recovery rates between 60 and 80%. Addition of a thioether group reduces the recovery rate to approximately 30%. The majority of ONPs have recovery rates around 90%. The addition of a nitro group with high polarity reduces the recovery rate to 10%. Five compounds with primary and secondary amines show recovery rates >200%. For these compounds, it is known that matrix effects could lead to unexpectedly high recovery rates. Compounds from the matrix can block active sites of the glass wool and liner, and an increased transfer to the GC column occurs [53]. For the majority of the compounds, the recovery rates were sufficient for these experiments. The LODs are: OPPs 1–250 pg, ONP 1–200 pg, HME 1–250 pg SPP 1–250 pg, SPP 2–100 pg on column. All LODs are listed in µg/L in the Table S2. These LODs correspond to 2.5 to 575 ng in 1 g of coffee beans. That is approximately 10 to 100 times higher compared to a described QuEChERS method [54]. The used sample preparation had the advantage of less chemical consumption, ease of use, and reduced sample preparation steps. Only 1 g of the coffee beans was used for the extraction with 5 mL organic solvent. The LODs can be easily improved by using more starting material and solvent evaporation.

**Figure 5:**
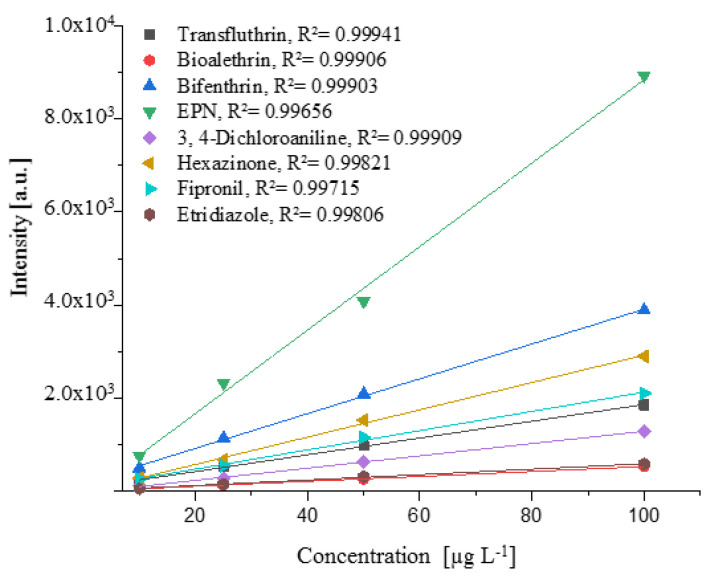
Relationship between concentration of the compound (µg L^−1^) and signal intensity (a.u.) for selected compounds of different pesticide classes. Linear regression curves were obtained with correlation coefficients R^2^ > 0.997.

The linearity was investigated between 10 and 100 µg L^−1^ and was demonstrated for organophosphorus, organonitrogen, herbicide methyl esters, and synthetic pyrethroid in Figure 5.

Figure 5 shows that a linear detector response was obtained with increasing concentration. For the suspected concentration of 10–100 µg L^−1^, correlation coefficients R^2^ > 0.997 were obtained, and the amount of pesticides can be determined by an external calibration.

A suspected target analysis of the plant protection products in Vietnamese coffee beans was done. The results are summarized in Table 2. For quantification of the compounds, an external calibration was used.

The coffee extract contains residues of herbicide methyl ester, organonitrogen, and organophosphorus compounds. Synthetic pyrethroide was not detected. These results are in agreement with most used plants protection products used in Vietnam [55]. One transient for each analyte was used for the dMRM method, and qualifier ions must be added to the method to improve the trueness of the results. The standard derivations for the three measured replicates were for all compounds ≤9% and were in the range for method validation and analytical quality control requirements published from the European commission of health and food safety (Document: SANTE/12682/2019). We were able to demonstrate that the performance under real conditions was the same as for the standards used in the method’s optimization. We postulate that the ion source can be used for a variety of analytes with high variety of functional groups and matrices.

## 3. Materials and Methods

### 3.1. Chemicals and Solutions

Acenapthene (99%), benzophenone (ReagentPlus^®^, 99%), cuminaldehyd (≥98%), dimethylphthalate (≥99%), and methyldodecanoate (≥99.5%), as substances for the evaluation of the ion source parameters, were purchased from Sigma-Aldrich (Steinheim, Germany). A multicompound solution (MIX-1) was prepared in methanol (LC-MS Grade) from T. Baker (Giwice, Poland) in a concentration range from 0.1 to 1000 µg/L.

A multiresidue pesticide kit (cat 32562) was purchased from Restek GmbH (Bad Homburg, Germany). It contains organophosphorus (OPP: cat 32563, 32570, 32571), organonitrogen (ONP: cat 32565, 32566, 32567), synthetic pyrethroide (SPP: cat 32568) compounds and herbicide methyl esters (HME: Cat 32569) in toluene (each 100 µg/mL). A multicompound solution of organophosphorus and organonitrogen was prepared. All pesticides were diluted with toluene to a final concentration between 0.1 and 1000 µg/L. Nitrogen and Helium 99.999% were obtained from Air Liquide (Duesseldorf, Germany).

### 3.2. Instrumentation

An Agilent 7890B gas chromatograph equipped with a split/splitess injector, autosampler (G453A), syringe 10 µL (9301-0713) and splitless liner (5190-3165) was coupled to a 6495 triple quadrupole LC/MS system from Agilent Technologies (Santa Clara, USA) using the new developed GC-APCI ion source. GC separation was performed on a nonpolar fused silica column DB-5MS (30 m, 0.25 µm film thickness and 0.25 mm i.d.) purchased from Agilent Technologies Inc. (Santa Clara, USA). The analyses were operated in constant flow mode. The make-up gas flow was controlled by a mass flow controller from ALLBORG model 325656.

### 3.3. Extraction Method and Evaluation of the Extraction Protocol

Commercially available coffee beans from Vietnam were grinded five times for 10 s with an electrical mill. One gram was weighed into a 25 mL propylene tube and 10 mL toluene was added. The suspension was extracted in an ultrasonic bath for 10 min at 50 °C, followed by centrifugation for 5 min at 400 rpm. The organic layer was transferred to a new propylene tube. This was repeated twice and the organic layers were merged. Afterwards, the volume of extraction solvent was reduced with a nitrogen stream at room temperature to approximately 2 mL and refilled with toluene to an exact volume of 5 mL. The sample was stored at −20 °C until analysis. Blank samples were prepared without coffee beans using the same procedure.

For the determination of the recovery rates of the extraction protocol, 1 g of the milled coffee beans was spiked with 1 µg of OPP (Restek 32563, 325670), ONP (Restek 32565, 32567), HME (Restek 32569), and SPP (32568) standards, which were dissolved in 500 µL of toluene. The spiked coffee beans were dried overnight. Afterwards, the described protocol was used.

### 3.4. Analytical Methods

#### 3.4.1. Influence of the Ion Source Parameters on the Ionization Behavior

All gas chromatographic analyses for MIX-1 were performed in constant flow mode with 1.0 mL/min Helium. One microliter was injected at 250 °C with a split ratio of 1:10. The temperature was programmed from 50 °C (0.2 min) to 280 (10 min) at 10 °C/min. The transfer line temperature was set to 290 °C. The QqQ-MS was operated in dynamic MRM mod (dMRM) with a retention time window of ±1 min. The product ions were determined by 10 collision energies (CE) between 10 and 70 eV, and the transient with the highest intensity was used for the dMRM method. As optimized APCI conditions, 200 °C ion source temperature, corona current 1 µA, needle height 2 mm, transfer capillary voltage 250 V, GC column position 2 mm, 1 mL min^−1^ nitrogen make-up gas flow and no additional water vapor were applied. The influence of these parameters was evaluated by altering one variable at a time and each time three replicates (*n* = 3) were injected. For the interday precision of the system five replicates (*n* = 5) were done.

#### 3.4.2. Application of Plant Protection Products in Coffee Beans

For the determination of plant protection reagents, the QqQ/MS was operated in positive ion dMRM mode with 3 min retention time window (RT ± 3 min). Therefore, the product ions of the plant protection products were determined by 10 CEs between 10 and 80 eV, and the transient with the highest intensity was used for the final dMRM method. As APCI conditions 200 °C ion source temperature, needle current 1 µA, needle height 2 mm, transfer capillary voltage 250 V, GC column 2 mm and no additional water vapor were applied. For the gas chromatographic separation, three different methods were used. Two microliters of the sample were injected in splitless mode at 250 °C. The transfer line was for all measurements 290 °C. For the determination of organophosphorus and organonitrogen compounds, the temperature program started at 50 °C (1 min) and was ramped up to 300 °C (3 min) at 5 °C/min. For herbicide methyl esters the temperature program started at 75 °C (1 min) and was ramped to 330 °C (10 min) at 20 °C/min. For the gas chromatographic separation of pyrethroide, the temperature program started at 100 °C (1 min) and was ramped to 150 °C at 25 °C/min. Afterwards, 150 °C was ramped to 300 °C (3 min) at 10 °C/min.

## 4. Conclusions

The developed GC-APCI ion source indicates a high potential for improving the sensitivity and repeatability of GC-APCI-MS coupling. The prototype ion source has an analytical performance with LODs between 0.5 to 250 pg on column for a broad range of compounds with different polarity, functional groups, and structure, which is in the range of commercially available APCI ion sources. These values were determined with the “increased accuracy mode” at the cost of sensitivity, because of the nonhumidified gas composition and low-pressure conditions in the ion source. Improvement of the gas supplies or reduced power of the pumping stage of the MS could increase the sensitivity significantly. The obtained repeatability for all compounds was independent on the concentration and displayed RSD < 10%.

Humidity and pressure in the ion source were determined as the main factors to increase the sensitivity of the introduced GC-APCI-MS system. The column position shows an impact on the amount of wall effects and hence repeatability and sensitivity. In contrast, temperature and needle height have shown only a slight impact on the ionization yields and repeatability.

The developed analytical method was successfully used for the suspected target screening of plant protection products in a Vietnamese coffee. Plant protection residues of OPP, ONP, and HME compounds were found.

## Figures and Tables

**Table 1:** Limit of detection (LOD) of the standards determined by the Kaiser and Specker approach.

Name	LOD Solution	Injection	Split	Mass on Column
	(nM)	(µL)		(pg)
Acenaphthene	30	1	10	0.5
Benzophenone	130	1	10	2.5
Cuminaldehyde	170	1	10	2.5
Diethylphthalate	40	1	10	1.0
Methyldodecanoate	50	1	10	1.0

**Table 2:** Hits of plant protection compounds in the used coffee sample with values of peak height (a.u.) from three replicates, average, relative standard derivation (RSD), and the final concentration in 1 g of the coffee beans.

Class	Name	M1 (a.u.)	M2 (a.u.)	M3 (a.u.)	Mean (a.u.)	RSD (%)	Concentration (ng/g)
HME	Metalaxyl	240	250	210	233	8.9	25
ONP	Paclobutrazol	310	280	290	293	5.2	239
OPP	Edifenphos	410	360	430	400	9.0	160
OPP	Fonofos	420	440	460	440	4.5	150
OPP	Sulprofos	190	165	180	178	7.0	376

## Supplementary Materials

The following are available online at https://www.mdpi.com/1420-3049/25/14/3253/s1, Figure S1: Developed prototype GC-APCI ion source. In S1A the optimum column position at 2 mm and the position of the GC column and corona needle in relation to each other. S1B shows the coupled system and the addition of the thermo couple, Figure S2A–D: Comparison of the detector response (a.u.) for pesticides with and without water addition to the make-up gas, Figure S3: Overlaid EICs for OPP standards from Restek 32563; 32570 and 32571 at 1 mg L^−1^. GC-Method: 50 (1min) to 330 °C (2 min), 5 °C/min; transfer line: 290 °C, injector: 250 °C; injection: 1 µL; constant flow: 1 mL/min He, Figure S4: Overlaid EICs for ON standard from Restek 32565; 32566, 32567 at 1 mg L^−1^. GC-Method: 50 (1 min) to 330 °C (2 min); 5 °C/min; transfer line: 290 °C; injector: 250 °C; injection: 1 µL; constant flow: 1 mL/min He. Figure S5: Overlaid EICs for SPP standard from Restek 32568 at 1 mg L^−1^. GC-Method: 100 (1min) to 150 °C; 25 °C/min; 150 to 300 °C (3 min); 10°C/min; transfer line: 290 °C; injector: 250 °C; Injection: 1 µL; constant flow: 1 mL/min He, Figure S6: Overlaid EICs for HME standard from Restek 32569 at 1 mg L^−1^. GC-Method: 100 (1 min) to 150 °C; 25 °C/min; 150 to 300 °C (3 min); 10 °C/min; transfer line: 290 °C; injector: 250 °C; Injection: 1 µL; constant flow: 1 mL/min He, Table S1: dMRM-Method Information for OPP, Table S2: dMRM-Method Information for ONP, Table S3: dMRM-Method Information for HME, Table S4: dMRM-Method Information for SPP.
