# Supplementary material for: Development of an Atmospheric Pressure Chemical Ionization Interface for GC-MS
Source: Molecules. 2020 Jul 16;25(14):3253. doi: 10.3390/molecules25143253 (PMC7397239; doi:10.3390/molecules25143253)
Supplement: Supplementary file 1 [file molecules-25-03253-s001.pdf]

molecules

MDPI

Supporting information

# Development of an Atmospheric Pressure Chemical Ionization Interface for GC-MS

**Christian Lipok, Florian Uteschil and Oliver J. Schmitz ***

University of Duisburg-Essen, Applied Analytical Chemistry, Universitaetsstr. 5, 45141 Essen, Germany; christian.lipok@uni-due.de (C.L.); florian.uteschil@uni-due.de (F.U.)

* Correspondence: oliver.schmitz@uni-due.de; Tel.: +49-201-183-3950; Fax: +49-201-183-3951

Academic Editor: Tomasz Tuzimski

## 1. GC-APCI-QqQ-coupling

The model of the prototype GC-APCI-MS is shown in **Figure S1**A and S1B.

A. Schematically drawing of the GC-APCI ion source with:

1. Make-up gas connection
2. Corona needle
3. GC column
4. Transfer line

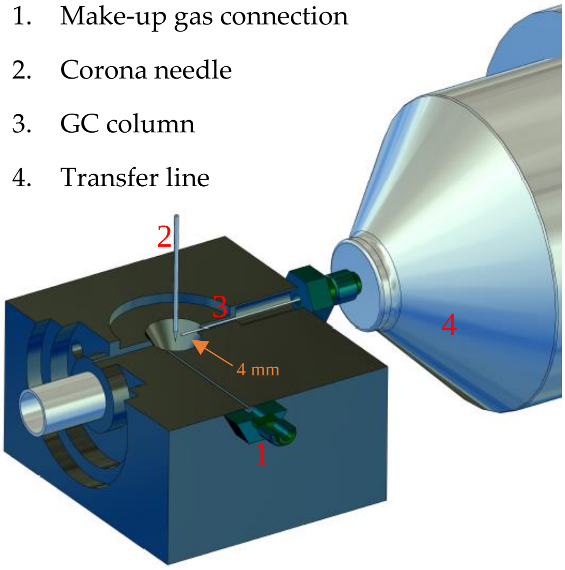


B. Realized coupling of the GC-APCI ion source with the 6495B QqQ form Agilent

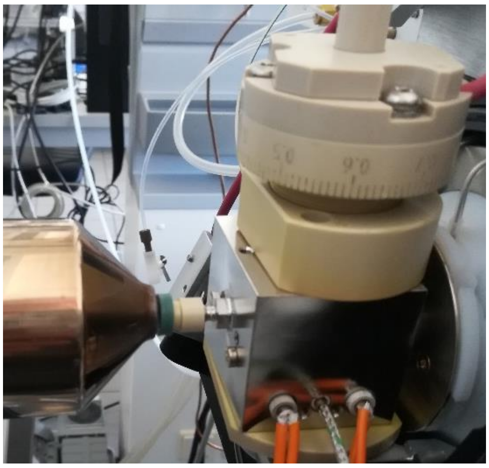

Thermo couple

**Figure S1**. Developed prototype GC-APCI ion source. In S1A the optimum column position at 2 mm and the position of the GC column and corona needle in relation to each other. S1B shows the coupled system and the addition of the thermo couple.

## 2. Pesticide Residues

### *2.1. Humidity*

The dependence of the detector response as a function of the make-up gas humidity is plotted in Figure S2A-F and shows that for almost all pesticides the detector response (Intensity in a.u.) are increased with the humidity. For all experiments 3 (n=3) replicates were measured.

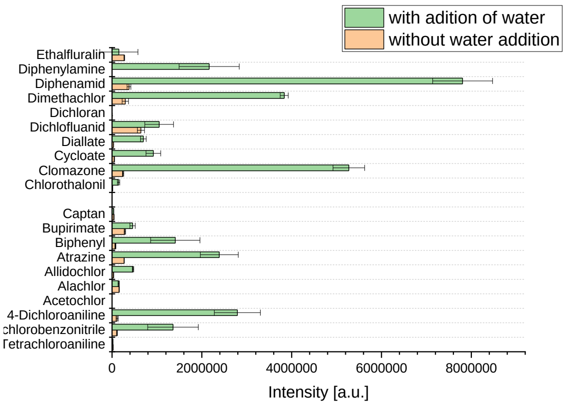


A

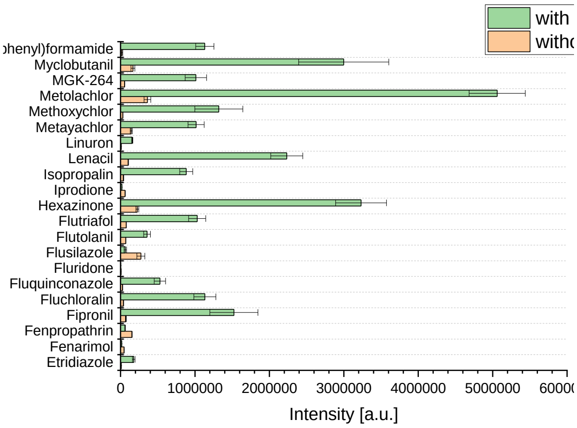


B

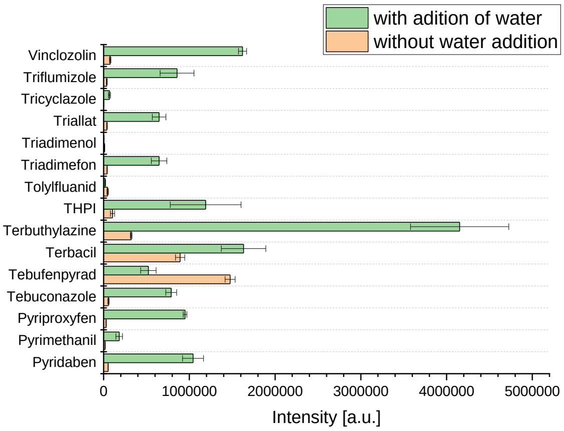


C

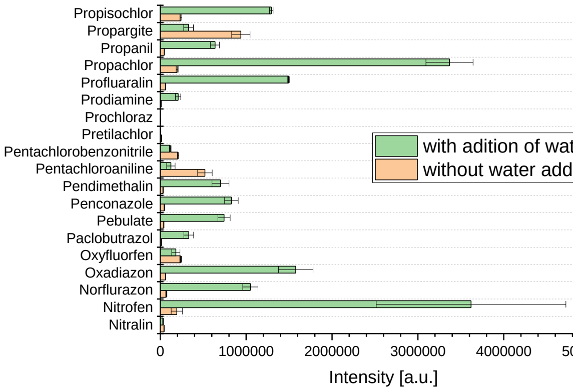


D

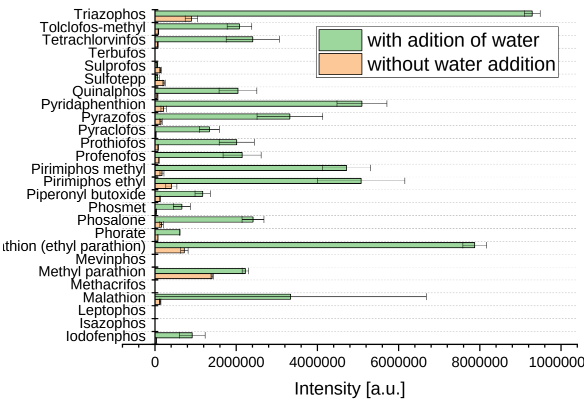


E

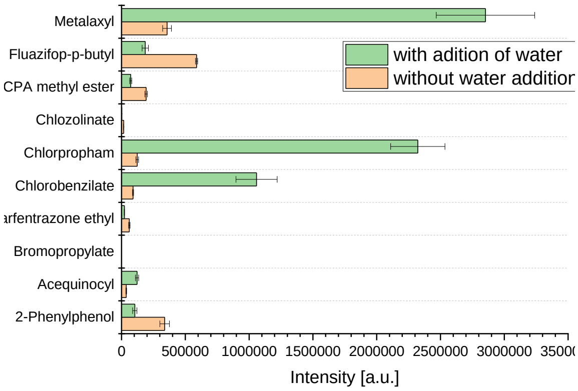


F

**Figure S2A–D**. Comparison of the detector response (a.u.) for pesticides with and without water addition to the make-up gas.

*2.2. dMRM-Method*

The dMRM-method are summarized in Table S 1, Table S2 and Table S3. The retention time, transient, collision energy, recovery rates and LOD of the compounds are shown. The corona needle was set to 1 µA and the transfer capillary to 250 V. The ion source was operated at 200 °C without humidified make-up gas (Nitrogen). Substances with (–) showing LODs higher than 500 g $L^{-1}$. For all substances 2 µL were injected in splitless mode at 250 °C.

**Table S1**. dMRM-Method Information for OPP.

| Name | Retention time min | Precursor m/z | Product Ion m/z | Energy V | Recovery % | Lod µg/L |
|---|---|---|---|---|---|---|
| Azinphos ethyl | 41.63 | 346.1 | 132 | 10 | 55.3 | 25.0 |
| Azinphos methyl | 31.4 | 318 | 131.9 | 10 | 40.1 | 125 |
| Bromfenvinfos-methyl | 32.46 | 377 | 126.9 | 5 | 52.2 | 12,5 |
| Bromfenvinphos | 34.01 | 405 | 127 | 20 | 37.1 | 12,5 |
| Bromophos ethyl | 33.17 | 395 | 338.7 | 20 | 56.0 | 12,5 |
| Bromophos methyl | 31.49 | 367 | 124.8 | 20 | 73.5 | 12,5 |
| Carbophenothion | 36.88 | 343.1 | 157.8 | 20 | 34.8 | 125 |
| Chlorfenvinphos | 32.54 | 359.1 | 155 | 10 | 54.3 | 25.0 |
| Chlorpyrifos | 30.92 | 350 | 197.8 | 20 | 49.9 | 5.00 |
| Chlorpyrifos methyl | 28.96 | 324 | 124.9 | 20 | 81.8 | 1,25 |
| Chlorthiophos | 36.3 | 361 | 326 | 10 | 169.5 | - |
| Coumaphos | 43.1 | 363.1 | 108.9 | 20 | 87.5 | - |
| Diazinon | 27.3 | 305.2 | 153.1 | 20 | 56.7 | 1.25 |
| Edifenphos | 36.9 | 311.1 | 109 | 30 | - | 25.0 |
| EPN | 39.16 | 324.1 | 296 | 10 | 35.0 | 1.25 |
| Fenchlorphos | 29.52 | 321 | 124.9 | 30 | 80.2 | 50.0 |
| Fenitrothion | 30.13 | 278.1 | 124.9 | 20 | 71.0 | 0.50 |
| Fenthion | 30.86 | 279.1 | 108.9 | 20 | 63.3 | 25.0 |
| Fonofos | 25.85 | 247.1 | 109.1 | 20 | - | 1.25 |
| Iodofenphos | 33.97 | 413 | 124.7 | 30 | 64.9 | 50.0 |
| Leptophos | 33.94 | 413 | 170.9 | 20 | 65.6 | - |
| Malathion | 30.64 | 331.1 | 127 | 10 | 23.4 | 0.50 |
| Methyl parathion | 29.29 | 264.1 | 109.1 | 20 | 50.2 | 1.25 |
| Phosalone | 40.42 | 368.1 | 181.9 | 10 | 39.8 | 5.00 |
| Phosmet | 39.00 | 318 | 159.9 | 5 | 64.8 | 12.5 |
| Pirimiphos ethyl | 31.91 | 334.2 | 198 | 20 | 75.1 | 1.25 |
| Pirimiphos methyl | 30.26 | 306.2 | 164 | 20 | 41.6 | 2.50 |
| Profenofos | 34.26 | 375.0 | 304.8 | 20 | 58.3 | 1.25 |
| Prothiofos | 34.1 | 345.0 | 240.8 | 20 | 42.4 | 12.5 |

| | | | | | | |
|---|---|---|---|---|---|---|
| Pyraclofos | 42.02 | 361.1 | 256.9 | 20 | 31.5 | 50.0 |
| Pyrazofos | 41.77 | 374.2 | 222.2 | 20 | 43.1 | 5.00 |
| Pyridaphenthion | 39.03 | 341.1 | 189 | 20 | 37.7 | 25.0 |
| Quinalphos | 32.61 | 299.2 | 147 | 20 | 49.3 | - |
| Sulfotepp | 24.81 | 323.2 | 145.9 | 30 | 39.8 | 100 |
| Sulprofos | 36.55 | 323.1 | 96.6 | 30 | - | 125 |
| Tetrachlorvinfos | 33.53 | 367 | 126.9 | 20 | 60.3 | - |
| Tolclofos-methyl | 29.10 | 301 | 124.9 | 20 | 71.6 | 0.5 |
| Triazophos | 26.80 | 314.2 | 162 | 20 | - | 25 |

**Table S2**. dMRM-Method Information for ONP.

| Name | Retention time<br>min | Precursor<br>m/z | Product Ion<br>m/z | Energy<br>V | Recovery<br>% | Lod<br>μg/L |
|---|---|---|---|---|---|---|
| 2. 3. 5. 6-Tetrachloroaniline | 23.45 | 231.4 | 195.7 | 20 | 200.0 | 25.0 |
| 2. 6-Dichlorobenzonitrile | 16.83 | 172 | 136 | 30 | 312.5 | 2.50 |
| 3. 4-Dichloroaniline | 19.16 | 162 | 127 | 20 | 7.8 | 50.0 |
| Atrazine | 26.26 | 216.1 | 174 | 20 | 106.1 | 2.50 |
| Biphenyl | 17.7 | 155.1 | 77.1 | 30 | 117.8 | 50.0 |
| Bupirimate | 35.05 | 317.2 | 209.1 | 20 | 71.9 | 2.50 |
| Dichlofluanid | 30.27 | 223.9 | 122.9 | 20 | 119.1 | 5.00 |
| Dimethachlor | 28.61 | 256.1 | 224.1 | 20 | 120.0 | 0.50 |
| Diphenamid | 31.61 | 240.2 | 134 | 30 | 145.5 | 2.50 |
| Diphenylamine | 23.66 | 170.1 | 92.1 | 20 | 105.8 | 5.00 |
| Ethalfluralin | 24.34 | 334.2 | 317.2 | 5 | 64.5 | 12.5 |
| Etridiazole | 19.38 | 248.9 | 220.8 | 5 | 135.7 | 0.50 |
| Fenarimol | 41.3 | 331.1 | 139 | 5 | 110.0 | 50.0 |
| Fipronil | 32.82 | 437.1 | 367.8 | 20 | 96.9 | 2.50 |
| Fluchloralin | 27.53 | 356.2 | 314 | 10 | 75.0 | 0.50 |
| Flutriafol | 33.72 | 302.1 | 70 | 20 | 91.7 | 12.5 |
| Hexazinone | 37.76 | 253.2 | 171 | 10 | 75.0 | 2.50 |
| Lenacil | 37.23 | 235.1 | 153 | 20 | 71.2 | 12.5 |
| MGK-264 | 31.48 | 276.1 | 210.1 | 10 | 58.7 | 2.50 |
| Myclobutanil | 34.79 | 289.2 | 70.1 | 20 | 124.3 | 2.50 |
| Oxadiazon | 34.71 | 345.1 | 219.9 | 20 | 77.4 | 0.50 |
| Paclobutrazol | 33.25 | 294.2 | 276.1 | 10 | - | 25.0 |
| Pebulate | 19.55 | 204.1 | 128.1 | 10 | 90.0 | 2.50 |
| Penconazole | 32.25 | 284.1 | 70 | 30 | 32.0 | 100 |
| Pendimethalin | 32.13 | 282.1 | 211.9 | 5 | 15.3 | 0.5 |
| Prodiamine | 30.4 | 351.2 | 246.9 | 20 | 7.0 | 2.50 |
| Profluralin | 27 | 348.2 | 232 | 10 | 4.2 | 0.5 |
| Propargite | 38.08 | 350.2 | 201 | 10 | 70.7 | 5.0 |
| Pyrimethanil | 27.27 | 200.1 | 82.2 | 20 | 177.6 | 12.5 |
| Pyriproxyfen | 40.81 | 322.2 | 95.9 | 30 | 68.4 | 100 |

| | | | | | | |
|---|---|---|---|---|---|---|
| Tebuconazole | 37.81 | 308.1 | 70 | 20 | 94.3 | 12.5 |
| Terbacil | 27.71 | 161 | 144 | 20 | 81.3 | 12.5 |
| Terbuthylazine | 26.75 | 230.1 | 174 | 20 | 85.0 | 2.5 |
| Tetrahydrophthalimide | 20.15 | 152.2 | 81.1 | 10 | 78.0 | 5.0 |
| Triadimefon | 31.09 | 294.2 | 224.9 | 10 | 64.6 | 2.50 |
| Tricyclazole | 35.13 | 190.1 | 163 | 20 | 88.9 | - |
| Triflumizole | 33.12 | 346.1 | 278 | 5 | 66.2 | 12.5 |
| Vinclozolin | 29.06 | 286 | 241.9 | 10 | 108.4 | 0.50 |

**Table S3**. dMRM-Method Information for HME.

| Name | Retention time<br>min | Precursor<br>m/z | Product Ion<br>m/z | Energy<br>V | Recovery<br>% | Lod<br>µg/L |
|---|---|---|---|---|---|---|
| 2-Phenylphenol | 6.98 | 171 | 142 | 30 | 164.7 | - |
| Chlorpropham | 7.7 | 172 | 154 | 10 | 86.5 | 0.50 |
| Metalaxyl | 9.8 | 280 | 220 | 10 | - | 0.50 |
| DCPA methyl ester | 9.52 | 333 | 301.9 | 20 | 64.8 | 125.0 |
| Chlozolinate | 9.82 | 332.1 | 186.9 | 20 | 84.7 | 25.0 |
| Chlorobenzilate | 10.5 | 307.2 | 250.9 | 20 | 64.7 | 2.50 |
| Acequinocyl | 12.85 | 343.3 | 188.9 | 20 | 176.5 | 12.5 |

**Table S4**. dMRM-Method Information for SPP.

| Name | Retention time<br>min | Precursor<br>m/z | Product Ion<br>m/z | Energy<br>V | Recovery<br>% | Lod<br>µg/L |
|---|---|---|---|---|---|---|
| Allethrin | 10.4 | 303.2 | 135.0 | 10 | 64 | 20 |
| Bifenthrin | 13.8 | 181.0 | 165.0 | 30 | 62 | 2 |
| Cypermethrin | 16.5 | 416.1 | 191.0 | 10 | 69 | 10 |
| Deltamethrin | 17.9 | 504.0 | 278.8 | 10 | 69 | 100 |
| Tetramerthrin | 13.8 | 332.2 | 164.0 | 30 | 129 | 20 |
| Transfluthrin | 8.8 | 371.0 | 163.0 | 20 | 69.9 | 20 |
| Phenothrin | 14.2 | 351.2 | 183.0 | 20 | 64 | 100 |

Overlaid EICs of the analyzed pesticides standards (1 mg $L^{-1}$ in toluene) from Restek are shown in Figure S 3 OPP, Figure S 4 ONP, Figure S5 SPP and Figure S6 HME.

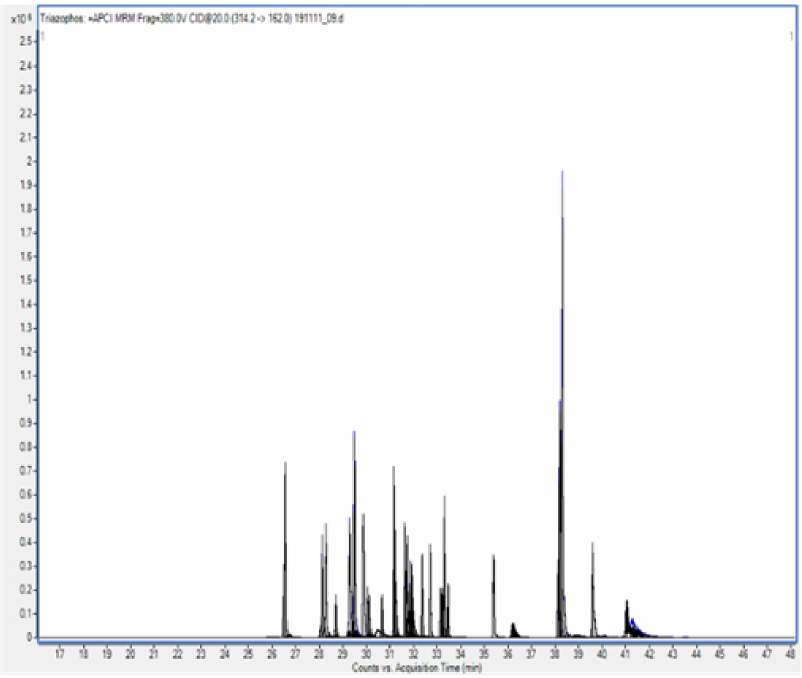


**Figure S3**. Overlaid EICs for OPP standards from Restek 32563; 32570 and 32571 at 1 mg L$^{-1}$. GC-Method: 50 (1min) to 330 °C (2 min), 5 °C/min; transfer line: 290 °C, injector: 250 °C; injection: 1 µL; constant flow: 1 mL/min He.

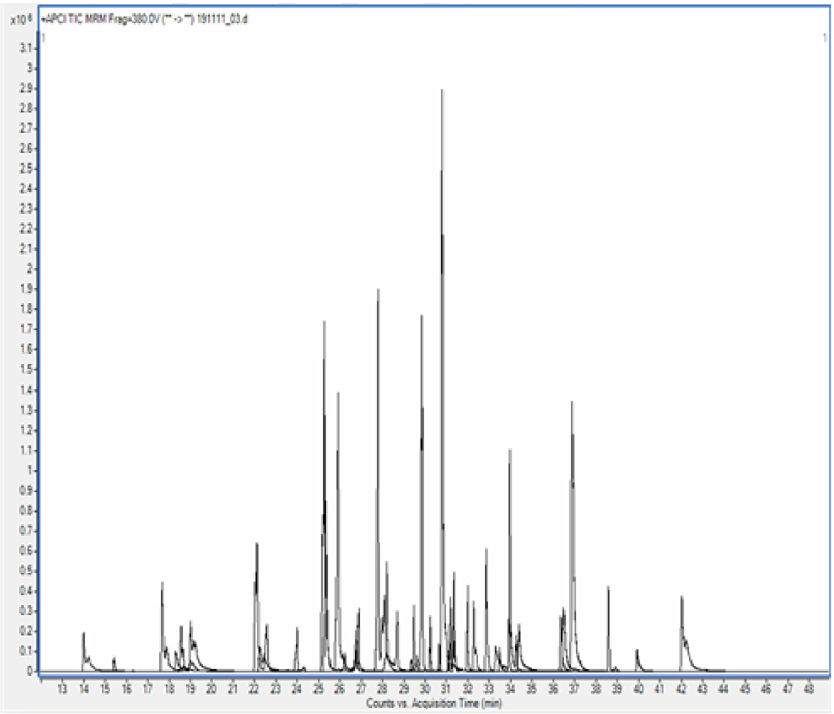


**Figure S4.** Overlaid EICs for ON standard from Restek 32565; 32566, 32567 at 1 mg L$^{-1}$. GC-Method: 50 (1 min) to 330 °C (2 min); 5 °C/min; transfer line: 290 °C; injector: 250 °C; injection: 1 µL; constant flow: 1 mL/min He.

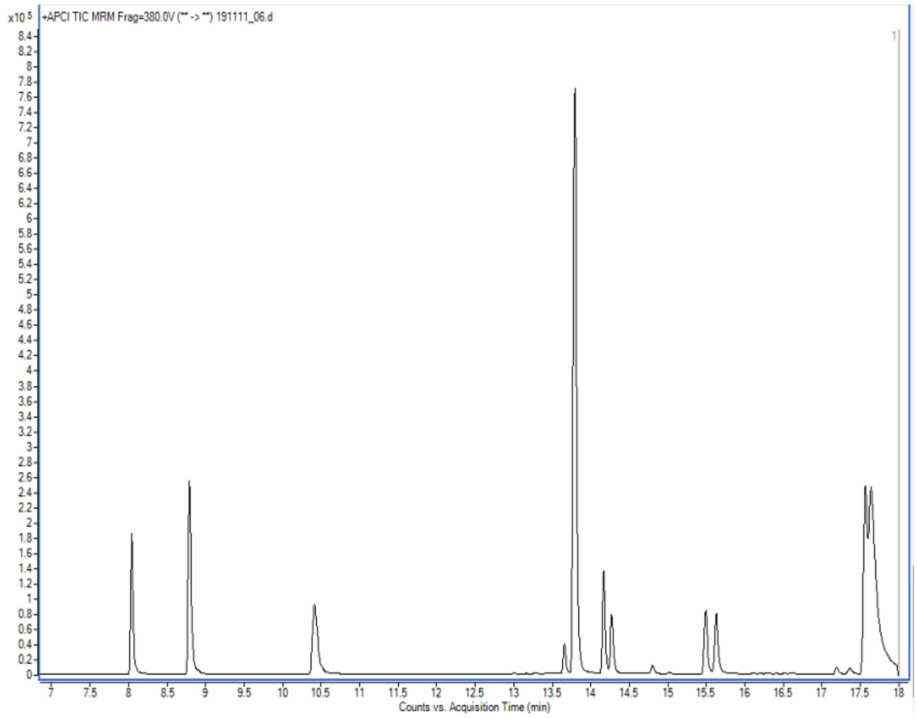


**Figure S5.** Overlaid EICs for SPP standard from Restek 32568 at 1 mg L$^{-1}$. GC-Method: 100 (1min) to 150 °C; 25 °C/min; 150 to 300 °C (3 min); 10°C/min; transfer line: 290 °C; injector: 250 °C; Injection: 1 µL; constant flow: 1 mL/min He.

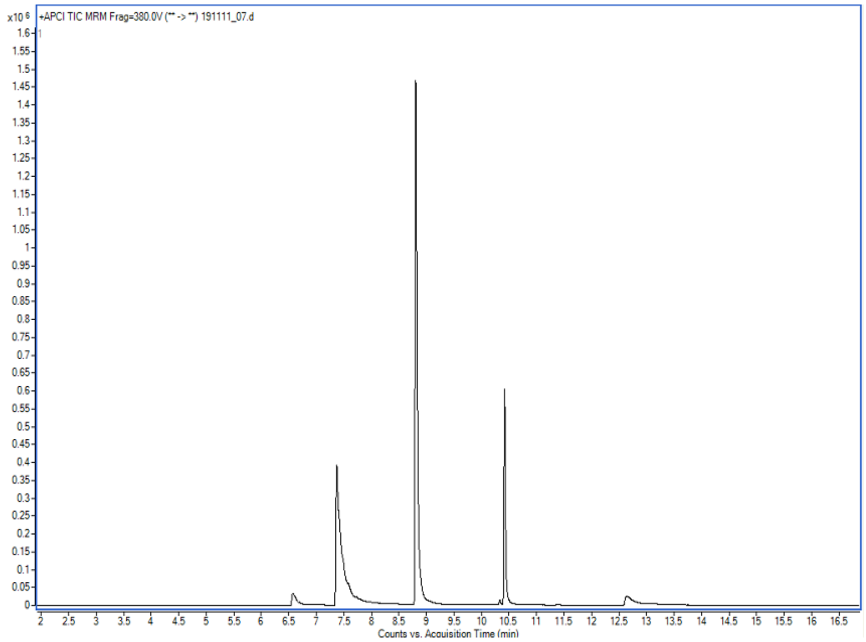


**Figure S6.** Overlaid EICs for HME standard from Restek 32569 at 1 mg L$^{-1}$. GC-Method: 100 (1 min) to 150 °C; 25 °C/min; 150 to 300 °C (3 min); 10 °C/min; transfer line: 290 °C; injector: 250 °C; Injection: 1 µL; constant flow: 1 mL/min He.
